# On the Timing of Marriage and Childbearing: Family Formation Pathways Among Immigrants in Switzerland

**DOI:** 10.1007/s10680-024-09702-w

**Published:** 2024-05-22

**Authors:** Julie Lacroix, Júlia Mikolai, Hill Kulu

**Affiliations:** School of Geography and Sustainable Development, University of St Andrew, St Andrews, UK

**Keywords:** Second demographic transition, Immigrants, Fertility, Marriage, Nonmarital childbearing

## Abstract

This paper examines childbearing in and outside of marriage as a manifestation of the Second Demographic Transition among immigrant populations in Switzerland. Based on full-population register data, we simultaneously analyse fertility and partnership changes at different stages of the migration process. Results from a multistate event history model show that most of the differences in family formation patterns between migrant groups and natives are in the sequencing of marriage and first birth among childless unmarried women. Out of wedlock family trajectories prove to be a common experience for European migrants, but a sustainable family pathway only among natives, as well as among immigrants from France, and Sub-Saharan Africa. Among married women, it is the risk of a third birth that marks the differences between groups; first and second birth rates are relatively similar across migrant groups. Distinguishing between the transition patterns of newly arrived immigrants and settled immigrants (characterised by various residence durations) support the disruption hypothesis among EU migrants and the interrelated life events hypothesis among non-EU groups. Family size and the partnership context of fertility highlight which family regime prevails in different population subgroups and the role that immigrants play in the Second Demographic Transition and family transformation in Europe.

## Introduction

Europe has seen significant changes in family formation patterns and living arrangements since the 1950s (Buchmann & Kriesi, 2011). New forms of conjugal life and entry into adulthood have gradually become more common and acceptable. This includes the increase in (premarital) cohabitation, childbearing outside of marriage, divorce, or re-partnering. Changes in the type and timing of events, and the order in which they occur have been seen as a manifestation of the Second Demographic Transition (SDT) (Lesthaeghe, 2010, 2014). Nonetheless, research shows that instead of being a general trend that uniformly affects all individuals and all family life domains, variation in life courses took distinct forms and paces in different countries and among different social groups (Van Winkle, 2018; Widmer & Ritschard, 2009). In Europe, the growing share of international migrants from countries with different family systems contributes to this diversity (Andersson, 2021). However, despite extensive research on fertility and partnership dynamics among immigrant populations, the partnership context of fertility, including nonmarital or (pre)marital childbearing, only received passing attention (Adserà & Ferrer, 2015).

The literature proposes different views on why immigrants’ family behaviours differ from that of natives in host countries with the aim of understanding the consequences of migration for families. A large stream of research focuses on heterogeneity in fertility (Kulu et al., 2017, 2019) while another stream examines variation in partnership formation or dissolution, with a strong focus on intermarriage (Andersson et al., 2015; Hannemann & Kulu, 2015; Hannemann et al., 2020; Kulu & González-Ferrer, 2014). The literature highlights the influence of social distance and time (i.e. duration since migration/inter-generational change) in explaining the distinctiveness of the migrant population, generally through processes of socialisation and adaptation. In Europe, studies show greater differences in fertility and partnership patterns among immigrants from geographically (and culturally) distant countries, characterised by higher marriage than cohabitation rates, and higher third and fourth birth risks. Recently, joint examination of childbearing and partnership transitions has further revealed that changes in fertility behaviours across generations occur more rapidly than changes in partnership behaviours (Kulu et al., 2022; Mikolai & Kulu, 2022). While fertility decisions seem more affected by structural-economic factors and individuals’ life circumstances, partnership behaviours appear to be more influenced by normative cultural factors (Andersson, 2020; Kulu et al., 2022). Nevertheless, what is often perceived as family ideals or cultural preferences may be induced by the migration process itself, which involves strict legal requirements, especially in the family domain.

Using full-population register data, we examine the prevalence and sequencing of marriage and childbearing by parity among immigrant and native women in Switzerland. We enhance previous research on immigrant family life courses in several ways. First, we focus on childbearing by parity in and outside of marriage as a manifestation of the SDT for various migrant groups. The SDT focuses on interrelated changes in fertility, family formation, and partnership behaviour (Sobotka, 2008) induced by ideological and cultural shifts toward an “individualistic family model” (Lesthaeghe, 2010, 2014). Declining fertility rates (below replacement levels) and a weakening link between marriage and childbearing are key indicators of the STD; a useful framework to explain family formation trends among immigrants who may find themselves in-between two cultures. The adoption of family formation behaviours prevalent in the receiving country, including family size and union type, is often perceived as indicative of social distance between groups and assimilation to mainstream society (Holland & Wiik, 2021). Whether women marry first and then have children, marry largely after the first birth, marry later, or never marry (with or without having (additional) children) provide insights into the family norms and values of immigrants with various cultural backgrounds. The timing and prevalence of divorce is also indicative of prevailing family norms in certain groups and is included as a competing outcome in family trajectories.

Second, we distinguish newly arrived immigrants from settled immigrants (characterised by various residence durations) and emphasise family patterns that are interrelated with the migration event (i.e. the arrival effect) and those that occur later. Studies have shown how (international) migration and family formation are interrelated as they are often part of the same process (Milewski, 2007; Mulder, 1993). However, the interaction of family ideals, as expressed by the type and sequencing of events, and legal constraints (i.e. entry requirements imposed by migration policies) have received little attention. Legal requirements lead to a selection effect, and selection is expected to operate differently across origin countries depending on whether women have free movement and access to the labour market. The reason for migration clearly shapes family formation patterns with delayed family transitions for employment migrants and accelerated transitions for marriage migrants (Kulu et al., 2019). The level and sequence of family events may also be distorted by the act of migration, thus creating a mismatch with family ideals, e.g. by opting for marriage rather than a consensual union to secure legal status in the country. By studying the risk of first and higher order births among unmarried and married women at different stages of the migration process, we seek to distinguish between behaviours that are induced by (or happen in tandem with) the migration process and those that are the result of cultural norms or preferences.

Third, we use register data for the entire resident population of Switzerland for the period 2012–2018. The population register was adopted only recently, in 2010, and thus register-based (partnership and fertility) research is still very new in the country. The data document all births, marriages, and divorces for the entire resident population which allows for detailed group-specific analysis. We take advantage of this comprehensive data to highlight variations in family pathways of a large number of immigrant groups in a context where migration is mainly motivated by professional reasons but with important differences by origin. Finally, we consider the unique cultural diversity of the native Swiss population and compare the family formation patterns of immigrants from neighbouring countries (Germany, France, and Italy) with that of natives from respective linguistic regions.

Following new developments in family life course research (see Kulu et al., 2022; Mikolai & Kulu, 2022), we investigate partnership and fertility trajectories jointly in a multistate event history framework. We model the time to a set of competing events among unmarried and married women and analyse (1) the transition to marriage or a (first, second or third) birth among unmarried women, and (2) the transition to divorce or a (first, second or third) birth among married women. Simultaneous analysis of partnership changes and childbearing allows for a better understanding of which family pattern (as expressed by family size and partnership context of fertility) prevails in different population subgroups and the role that immigrants play in the SDT and family change in Europe.

## Background

### Migration, Partnership Changes, and Fertility: Theoretical Considerations

Five well-established hypotheses explain the differences in family formation patterns between natives and immigrants (for overviews see Kulu, 2005; Kulu & González-Ferrer, 2014). Some emphasise the influence of origin and destination contexts, while others highlight the role of the migration process itself, thus disentangling the role of structural factors from those of the cultural context in family behaviours (Andersson, 2020). The *socialisation hypothesis* emphasises cultural inheritances and the persistence of norms and values acquired early in life. Important decisions about family size and union type are expected to reflect the dominant family model at origin. Differences in family behaviours are thought to persist over time (since migration) and even be transmitted across migrant generations. The *adaptation hypothesis*, by contrast, states that immigrants will adapt their behaviours to the new social environment. With time and prolonged exposure to new family norms at the destination, the family behaviours of immigrants are expected to converge to that of the natives.

The *selection hypothesis* expects similarities in family behaviours between immigrants and natives due to immigrants choosing a destination that matches their preferences and lifestyle, including in the family domain. Immigrants are a select group; i.e. their preferences differ from the dominant norms in the country of origin and resemble those prevailing in the host country (Mikolai & Kulu, 2022). Legal requirements for migration also imply a selection effect. Unlike the self-selection described above, the selection induced by the legal entry requirements does not imply similarities with the native population but differences across migrant origin groups. Depending on the country of citizenship, access to the territory is limited to specific administrative grounds. While EU migrants in Switzerland enjoy freedom of movement and access to the labour market, access to visas for non-EU citizens is often limited to family purposes, and access to the labour market is subject to a strict quota system. As a result, the migration system contributes to the selection of family-oriented profiles among non-EU migrants and work-oriented ones among EU migrants.

The relevance of the disruption and interrelation of life events hypotheses is also related to migration motives and (family) circumstances. On the one hand, the *disruption hypothesis* highlights the uncertainty, stress, and integration challenges that surround the migration process. Family formation plans may be delayed until migrants have established themselves economically, socially, and culturally in the host country (Kulu et al., 2019). For couples, decreased fertility shortly before and/or after migration is expected, especially among partners who moved at different time periods and lived apart for some time (Milewski, 2007). For singles, one can expect delayed marriage and childbearing due to the time needed to find a partner. The *interrelation of life events*, on the other hand, states that migration coincides with other family events (Andersson, 2004). From a life course perspective, migration and family formation are seen as interdependent or “parallel careers” (Courgeau, 1990; Mulder & Wagner, 1993). Migration, marriage, and first pregnancy tend to follow each other closely and the transition to first birth is common during the first year of marriage (Baizán et al., 2003; Milewski, 2007). While the disruption hypothesis can be expected to be more relevant for single migrants and those migrating for employment reasons (especially for women), the interrelation of life events hypothesis may be more applicable to family migrants.

### Immigrants’ Family Behaviours: Empirical Evidence

Research on partnership dynamics in Europe has examined the timing, type, and likelihood of union formation and dissolution among immigrants. In the past, the partnership pathways of immigrants resembled those of the native populations in Europe. Despite higher fertility levels for some migrant groups both immigrants and natives tended to follow a path of direct marriage and childbearing within marriage (Mikolai & Kulu, 2022). Increased family complexity and diversity in the last decades, including the postponement of marriage and the spread of cohabitation, nonmarital childbearing, and divorce (Thomson, 2014), have been observed to varying degrees in the migrant population.

Recent studies have shown significant heterogeneity in partnership formation according to the origin of international migrants. In most cases, these behaviours seemed to reflect the patterns prevailing in immigrants’ countries of origin, providing support for the socialisation hypothesis (e.g. Hannemann et al., 2020; Kulu et al., 2022). Nevertheless, other studies have found evidence of adaptation and selection mechanisms (e.g. Andersson et al., 2015; González-Ferrer et al., 2016; Hannemann & Kulu, 2015; Pailhé, 2015; Rahnu et al., 2015). In general, studies show that immigrants from geographically and culturally close countries (e.g. EU migrants in Europe) have family patterns similar to those of the natives, including a higher propensity to cohabit before marriage or a first birth. By contrast, immigrants from more conservative countries often follow a path of direct marriage and have larger families (see Andersson et al., 2015 for Sweden; Delaporte & Kulu, 2022, Pailhé, 2015 for France; Kuhnt & Krapf, 2020, Liu & Kulu, 2021 for Germany; Mikolai & Kulu, 2022 for the UK).

Couple formation (or marriage migration) as a special case of family reunification largely account for the elevated marriage and first birth risks around international migration (Andersson et al., 2015; Toulemon, 2004). The interrelation of life events hypothesis is particularly relevant in explaining immigrants’ partnership patterns. Family-related migration remains a dominant form of legal entry for non-European immigrants across Europe. Although ways of living together as a family have changed (e.g. nonmarital cohabitation, living apart together), most countries still adhere to the traditional model of marriage as a basis for entry into the territory (Kofman, 2004). As a result, family ideals may be distorted by the intention to migrate to another country, with accelerated and elevated transitions to marriage for those whose visa is conditional on family ties. By contrast, single migrants were shown to marry at older ages (Carlson, 1985; Milewski, 2003)—a pattern that may be explained by a longer search for a partner (Milewski, 2007). This is in line with the disruption hypothesis. The postponement of marriage (and possibly parenthood) for unmarried migrants may also be explained by a selection effect: individuals migrating for education or employment may have different family aspirations or simply be at different stages of their life course.

Findings on family dissolution among immigrants are mixed. In a comparative study of four European countries, Hannemann and colleagues (2020) found lower divorce risks among women from more conservative countries (i.e. South Asian women in the UK and Turkish women in France) stressing the embeddedness of culture and social norms towards this event. Others have found higher divorce rates among (certain groups of) foreign-born individuals than among natives (Andersson, 2015, Nekby, 2012). The stress and disruption of family life induced by the migration process, and exposure to new gender norms were highlighted as possible explanations for relatively high divorce risks among immigrants. Exogamous marriage is another factor linked to a higher likelihood of divorce (Milewski & Kulu, 2014). However, divorce may be incompatible with maintaining legal status in the host country, thus diminishing the chances of experiencing this event especially in the first years after immigration.

Studies on fertility dynamics among immigrant populations are abundant (see Adserà & Ferrer, 2015; Kulu et al., 2019; Kulu & González-Ferrer, 2014; Kulu & Milewski, 2007 for reviews). Based on the five above-mentioned hypotheses, previous research has studied whether and how immigration influences fertility levels in European countries (Kulu & González-Ferrer, 2014). Again, studies report significant variation across population subgroups. While European immigrants often show similarities (or convergence) with natives, non-Western immigrants show higher levels of fertility. In general, immigrants from countries with more conservative family patterns experience earlier transitions to parenthood, and have similar risks of a second birth as natives, but the propensity of a third or fourth birth is higher among immigrants (Kulu et al., 2022). Higher fertility levels for non-Western immigrants were found among Eastern and Southern European immigrants in Switzerland (Rojas et al., 2018); Turkish and Sub-Saharan African immigrants in France (Delaporte & Kulu, 2022; Pailhé, 2015); Turkish immigrants in Germany (Milewski, 2007, 2010); immigrants from the Maghreb region in Spain (González-Ferrer et al., 2016); immigrants from Morocco and Turkey in Belgium (Van Landschoot et al., 2017); and Pakistani and Bangladeshi immigrants in the UK (Kulu & Hannemann, 2016; Kulu et al., 2017). Age at migration, marital status, and the reason for migration are strong predictors of the timing and levels of fertility (Andersson, 2004; Cygan-Rehm, 2011; Milewski, 2007; Wolf, 2016). Women who were married at the time of migration or migrated for family reasons had particularly high fertility levels whilst employment-related migrants had lower fertility levels during the first years after arrival (Castro Martin & Rosero-Bixby, 2011; Mussino & Strozza, 2012; Mussino et al., 2015; Persson & Hoem, 2014).

The literature on immigrants’ fertility and partnership dynamics has focused only marginally on the partnership context of fertility including nonmarital and (pre)marital childbearing (Adserà & Ferrer, 2015). By jointly analysing partnership and childbearing changes, a few studies recently addressed this gap showing a stronger association between marriage and childbearing, and a lower risk of nonmarital and premarital childbearing among immigrant populations (Delaporte & Kulu, 2022; Liu & Kulu, 2021). In a British study, Mikolai and Kulu (2022) concluded that European and Western immigrants are experiencing increasingly diverse family trajectories with cohabitation, nonmarital childbearing, and separation being common experiences. By contrast, the partnership pathways leading to childbearing among other immigrant groups have remained relatively stable over time. In a cross-national study, Kulu et al. (2022) found striking similarities in the preference for marriage across migrant origins with strongly marriage-centred family forms. They concluded that, compared to fertility behaviours, partnership patterns are less affected by the destination context and more by the migration background.

### Swiss Context

#### Migration Policy and Immigrant Population

As a country with a high standard of living and a dynamic labour market, Switzerland attracts a significant number of immigrants. The foreign-born population (i.e. the population born abroad regardless of nationality) represents 31% of the total population (SFSO 2021). Since 2002 and the ratification of the EU/EFTA Agreement of the Free Movement of Persons by Switzerland, the conditions of entry, residence, and work are facilitated for EU/EFTA nationals. Immigrants from EU member states (Germany, France Portugal, Italy, and Spain in the lead) account for about two-thirds of the migrant population. At the same time, entry for third-country nationals (Kosovo and Turkey are the most represented) was restricted to family reunification, study, and asylum (Piguet, 2005). Employment-related migration for third-country nationals is now limited by strict quotas to highly skilled workers. International organisations are an important entry point for highly qualified third-country nationals. As a result of these policies, the reasons for migration and the skill composition of the population vary by origin country. While employment is the main reason for migration for many European migrants (72% for Germans, 71% for Italians, and 58% for Western European migrants), family reasons are most common among migrants from the Balkans (73%), South America (68%), and West Africa (61%). This pattern also varies substantially by gender: 55% of women and only 22% of men moved to Switzerland for family-related reasons (nccr—on the move, 2023). The composition (marital and parental status) of the migrant population at arrival reflects this dynamic (see Table 2 in Appendix).

#### Family Dynamics

Switzerland has undergone major changes in family formation and pathways to adulthood since the 1970s (Kellerhals & Widmer, 2007). However, the country differs from other European countries in several ways. First, de‐standardisation of life courses among younger cohorts shows a persisting gender divide: men typically have stable and linear occupational trajectories while women often go back and forth between part‐time employment and family care (Widmer & Ritschard, 2009). Switzerland has a rather conservative gender and family system characterised by a lack of institutional support for working mothers (Rossier et al., 2023), leading to or reinforcing the dominant one-and-a-half breadwinner model. By contrast, immigrant women to Switzerland were shown to either work full-time or be inactive. When employed, however, the number of hours worked is higher for immigrants than for natives (Lacroix & Vidal-Coso, 2018).

Second, Switzerland has the lowest level of long-term fertility in Europe (together with Germany and Austria); the TFR has been around 1.5 since the mid-1970s (Sobotka, 2011). The impact of immigrant fertility on the overall TFR is larger in Switzerland than in other European countries (Sobotka, 2008). In 2021, almost 40% of all births were to women of foreign nationality.^1^ However, despite a TFR that is about 0.5 higher than that of Swiss women, foreigners do not have larger families. In fact, Burkimsher et al. (2020) as well as the statistics from household registration (STATPOP) and the Families and Generations Surveys (FGS) indicate the opposite. Although Rojas and colleagues (2018) reported higher first birth risks among immigrants, especially from Eastern and Southern Europe, they found a lower transition rate to a second birth among all immigrant groups than among natives.

Third, although premarital cohabitation is common, marriage is more prevalent in Switzerland than in most Europe countries, and its link with childbearing remains particularly strong (Lesthaeghe, 2010). Couples in Switzerland often marry before or during first pregnancy, or a few months after the first birth (Charton & Wanner, 2001; Rossier & Legoff, 2005). By European standards, the birth rate outside marriage is very low (28% at the national level, compared to 62% in France, 58% in Portugal, and 48% in Spain) (Eurostat, 2020). This means that many immigrants to Switzerland come from countries that are further along in the SDT. Nonetheless, Switzerland is a de-centralised federal state with well-defined institutional and cultural divisions. The SDT is not evenly widespread across the country: vital statistics show that out-of-wedlock birth rate is 13 percentage points lower for natives living in the German-speaking region than for those living in the French- or Italian-speaking regions (authors’ own calculation based on Statpop 2019). Research on the partnership patterns of immigrants in Switzerland mainly focused on mixed marriages (see for instance Potarca & Bernardi, 2018). The type of union (consensual union or marriage) and the partnership context of fertility (nonmarital childbearing, single parenthood) among immigrants have not yet received much attention.

### Hypotheses

Based on the literature, Swiss context, and distinct migration policies for EU and non-EU migrants in the country, we derive the following hypotheses. First, we expect immigrants’ partnership context (and levels) of fertility to echo their origin country’s dominant norms and progression in the SDT (*socialisation hypothesis*). Compared to the native population (for whom the link between marriage and childbearing remains strong), immigrants from EU countries, and especially immigrants from France and Portugal, are expected to have lower marriage rates and a greater propensity to have children (first and higher order births) outside marriage. By contrast, we expect higher marriage rates and a greater propensity to have larger families within marriage among non-Western (especially Turkish) immigrants.

When considering socialisation mechanisms, scholars generally compare behaviours in origin and destination countries (e.g. TFR, employment rates) at the national level. We further contrast the family behaviours of Italian, German, and French immigrants with that of natives in the respective linguistic regions. Although we do not derive specific hypotheses for those exploratory findings, we expect these differences to be indicative of cultural influences, but also of the way in which the migration process alters these influences.

Second, EU and non-EU migrants in Switzerland are subject to different entry requirements selecting work-oriented profiles in the former case and family-oriented ones in the latter. Specific migration dynamics such as marriage migration and family reunification also select immigrants at specific life course stages, influencing family transitions, especially in the short-term. Distinguishing newly arrived immigrants from settled immigrants, we test for different arrival effects for EU and non-EU immigrants. We expect EU migrants to experience lower marriage and fertility transitions in the first two years following arrival to Switzerland *(disruption and selection hypotheses)*. By contrast, we expect non-EU migrants to experience higher marriage and fertility transitions in the first two years following arrival to Switzerland *(interrelation of life events and selection hypotheses)*. Distinct patterns for newly arrived immigrants are expected, but mainly among childless women (Milewski, 2007).

## Data and Methods

### Data

We use linked administrative registers that cover all residents of Switzerland between 2012 and 2018. The data comes from three different sources: (1) The Population and Household register, (2) the Vital register, and (3) the Social Security register. The Population and Household register (STATPOP) provides information on different demographic dimensions for all persons legally living in Switzerland (on the reference date of December 31 of each year). These characteristics include age, sex, (date of) marital status, nationality, country of birth, and year of arrival in Switzerland. Additionally, a household ID allows to identify co-residents of the same dwelling but does not document their relationship.^2^ This means that information on parental status and number of children in the household (parity) is not directly available in the dataset. For women who do not experience any childbirth during this period, we define parity as the number of children in the household whose age difference with the mother is between 15 and 45 years.^3^ For women who experience childbirth during the observation period, this information is directly retrieved from the Vital register (description below).

Fertility and partnership transitions are extracted from the Vital register (BEVNAT), which provides continuous and detailed information on childbirth, marriage, and divorce, including the links to the parents or the (ex-)partner. For each new birth, the register also documents parity and marital status at birth. Family events that took place abroad among individuals domiciled in Switzerland are also documented in the register. The data does not gather information on nonmarital cohabitation which means that both unpartnered individuals and those in a nonmartial union are referred to as ‘unmarried’. This has some implications for the ‘risk population’ under study, especially when comparing the family behaviours of childless unmarried women of immigrant and non-immigrant backgrounds. Immigrants for whom entry to Switzerland is conditional on or eased by family visas are likely to be at specific stages of their family and reproductive lives. The risk of a first birth or marriage differs among individuals who are in a consensual union or unpartnered. We interpret the results in light of specific migration dynamics (i.e. marriage migration, family reunification) and discuss the implications in the conclusion. Nevertheless, although childbearing within cohabitation and lone parenthood are two distinct family trajectories, both can be considered a manifestation of the SDT. In addition, we expect that most births among unmarried women occur in partnerships rather than among single women. Finally, the Social Security register (CdC) contains the annual income of all residents with a declared professional activity in Switzerland.

We analyse women between the ages of 15 and 45. We start observing women at different ages in 2012 (or later for those who migrated to Switzerland or reached age 15 between 2013 and 2017) until 2018, or until they reach age 45, emigrate, or die. We exclude women who were already divorced or had three or more children when first observed. Overall, the study population consists of 1,803,295 women, 41% of whom are born abroad.

### Analytical Strategy

**Fig. 1:**
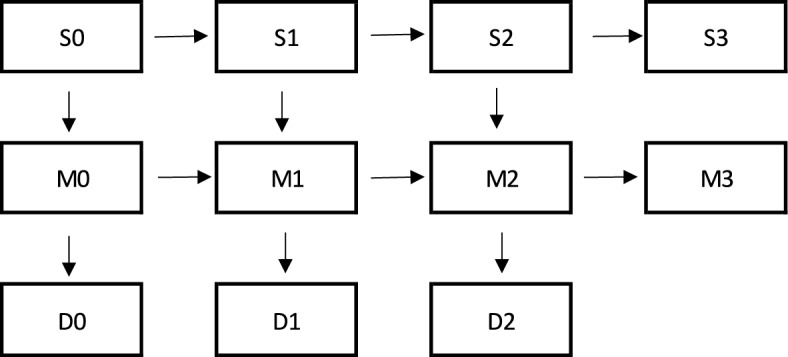
States and competing partnership and fertility outcomes. *Notes*: *S* Single (unmarried), *M* Married, *D* Divorce; the numbers 0–3 represent women’s parity (i.e. 0 child, 1 child, 2 children, or 3+ children)

To analyse partnership and fertility trajectories jointly, we use a multistate event history approach (see Mikolai & Kulu, 2022 for a comprehensive overview of the modelling strategy). Figure 1 illustrates the possible states (combinations of marital status and parity in boxes) and competing transitions (arrows) considered in this study. We estimate different sets of models by marital status (unmarried or married) and parity (childless, one-child, or two-child mothers). First, we analyse competing partnership and fertility outcomes for unmarried women. Unmarried childless women can either marry or have a first child; unmarried mothers with one child can either have a second child or marry; and unmarried mothers with two children can either have a third child or get married. In a second step, we examine the competing partnership and fertility outcomes for married women. This population is at risk of either having a (first, second, or third) child or experiencing a divorce.

We estimate piecewise constant exponential models for competing risks and incorporate different ‘clocks’. Age is the baseline risk in all models. In the risk sets of married women, we also account for marriage duration; in the risk sets of mothers, we account for time since last birth. An interaction term between the type of event and the migrant’s country of birth allows us to test whether certain groups are more likely to experience one transition than another.

### Variables

The main variable of interest is individuals’ country of birth. For all models, we compare the family behaviour of natives (born in Switzerland) with that of women born abroad. The descendants of immigrants (2nd generation) are grouped together with the native population as the data does not contain information on the parents’ country of birth. Register data for the entire resident population allows for country-specific analysis. We distinguish women born in Germany, Italy, France, Portugal, and Spain (the largest origin groups in Switzerland among EU countries), as well as women born in Kosovo and Turkey (the largest non-EU origin countries in Switzerland). Due to smaller cell sizes, we have grouped individuals born in other countries by region of birth, distinguishing women from other EU countries, other European countries (Macedonia, Serbia, Bosnia-Herzegovina, and Russia represent 78% of this groups), Sub-Saharan Africa, North Africa, Latin America, North America, South Asia, Asia, and Oceania. Throughout this paper we distinguish between EU and non-EU migrants, as the former are free to enter, live and work in Switzerland, while the latter are subject to strict legal requirements. Switzerland has well-defined institutional and cultural divisions, and the different linguistic regions share, to a certain extent, some similarities with their adjacent regions (France, Germany, and Italy). We exploit these differences as an additional layer of complexity and comment on the within group (i.e. natives from different linguistic regions) and between group (i.e. natives and immigrants from the same linguistic regions) differences in family formation patterns.

To capture the arrival effect, i.e. the interrelationship between the international migration and family formation decisions, we code individuals as ‘newly arrived immigrants’ during the first two-year period after arrival and as ‘settled immigrants’ the subsequent years. Age is categorised into 5-year age groups: 15–19 (reference), 20–24, 25–29, 30–34, 35–39, and 40–44. The models also include different duration variables. Marriage duration and time since last birth are divided into four categories: 0–1 year (reference), 1–3 years, 3–5 years, and 5+ years. The data does not contain information on the level of education. Instead, we use annual household income and the employment status of women as socio-economic indicators. Employment status is coded as employed if the woman received any income during the given year and not employed otherwise.

## Results

### Descriptive

Table 1 describes the number of person-years and family transitions by marital status and country of birth. Swiss women account for the largest share of person-years (63%) and events both as unmarried and married. All origin groups are in sufficient numbers to warrant detailed group-specific analysis. Approximately three quarters of the time at risk for native women is as unmarried. This proportion is higher than for all migrant groups and can be explained by the younger age structure of this population (individuals who reached age 15 between 2013 and 2018 were included in the dataset resulting in a larger population of those ages among the Swiss population). Other groups are underrepresented in the unmarried category. This is the case for (in ascending order) women born in Turkey, Kosovo, other European countries, North Africa, and South Asia. By contrast, women from Germany, France, and North America contribute to over 60% of their risk time as unmarried.

**Table 1:** Number of person-years and family events by marital status and country of birth, women aged 15–45 in Switzerland (2012–2019)

	Outcomes of single women				
	Person-years	Birth		Marriage	
		N	Rate	N	Rate
Switzerland	3,616,138	53,812	0.015	100,830	0.028
Italy	85,736	1,545	0.018	2,635	0.031
Germany	265,812	6,728	0.025	8,856	0.033
Portugal	109,087	3,771	0.035	4,048	0.037
France	121,614	3,313	0.027	2,937	0.024
Spain	36,772	894	0.024	1,134	0.031
Other EU countries	230,639	3,910	0.017	7,727	0.034
Kosovo	21,371	414	0.019	1,967	0.092
Turkey	16,447	146	0.009	888	0.054
Other European countries	97,397	1,522	0.016	6,513	0.067
Sub-Saharan Africa	72,948	4,026	0.055	2,188	0.030
North Africa	10,836	119	0.011	391	0.036
Latin America	83,963	1,850	0.022	2,544	0.030
North America	43,806	389	0.009	971	0.022
Asia	108,525	1,444	0.013	2,849	0.026
South Asia	24,986	458	0.018	1,017	0.041
Oceania	6,706	92	0.014	180	0.027

	Outcomes of married women				
	Person-years	Birth		Divorce	
		N	Rate	N	Rate
Switzerland	1,401,584	157,486	0.112	20,668	0.015
Italy	61,864	4,817	0.078	387	0.006
Germany	139,800	13,968	0.100	1,164	0.008
Portugal	169,720	10,649	0.063	967	0.006
France	59,244	5,282	0.089	617	0.010
Spain	25,554	2,047	0.080	183	0.007
Other EU countries	193,420	14,592	0.075	2,008	0.010
Kosovo	80,445	1,189	0.015	537	0.007
Turkey	63,937	4,909	0.077	879	0.014
Other European countries	301,988	26,376	0.087	3,119	0.010
Sub-Saharan Africa	62,851	5,886	0.094	1,213	0.019
North Africa	32,285	2,887	0.089	687	0.021
Latin America	138,049	7,954	0.058	2,784	0.020
North America	27,483	2,323	0.085	229	0.008
Asia	149,023	9,793	0.066	1,868	0.013
South Asia	69,105	4,894	0.071	334	0.005
Oceania	5131	408	0.080	53	0.010

*Sources*: Statpop, Bevnat (2012–2018). Authors’ own calculations

### Outcomes of Unmarried Women

**Fig. 2:**
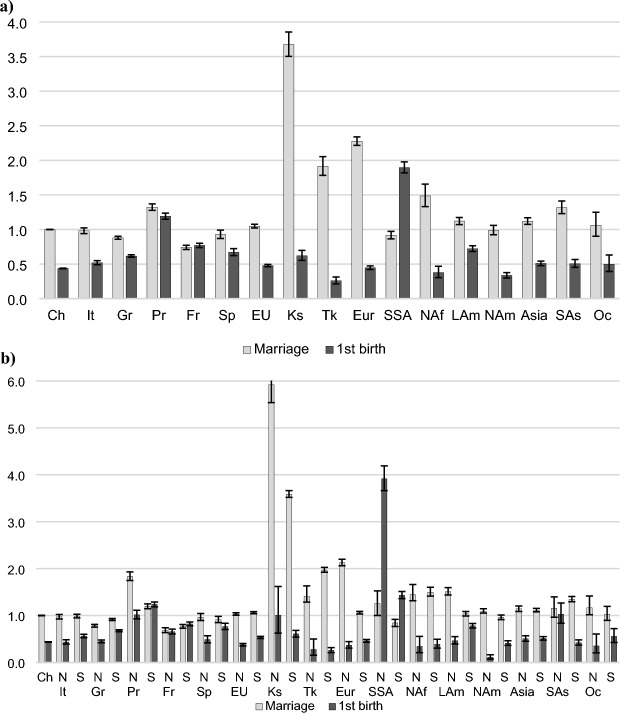
Outcomes of unmarried childless women. **a** Relative risks of a first birth or first marriage by migrant origin. **b** Relative risks of a first birth or first marriage by migrant origin, separately for newly arrived immigrants and settled immigrants. *Note*: *CH* Switzerland, *It* Italy, *Gr* Germany, *Pr* Portugal, *Fr* France, *Sp* Spain, *EU* other EU countries, *K*s Kosovo, *Tk* Turkey, *Eur* other European countries (not in the EU), *SSA* Sub-Saharan Africa, *NAf* North Africa, *Lam* Latin America, *Nam* North America, Asia, *SAs* South Asia, *Oc* Oceania. Control variables: age, employment status, household income. *N*  Newly arrived immigrants (first two years in the country), *S* Settled immigrants (subsequent years). *Sources*: Statpop, Bevnat, CdC (2012–2018). Authors’ own calculations. 95% confidence intervals

**Fig. 3:**
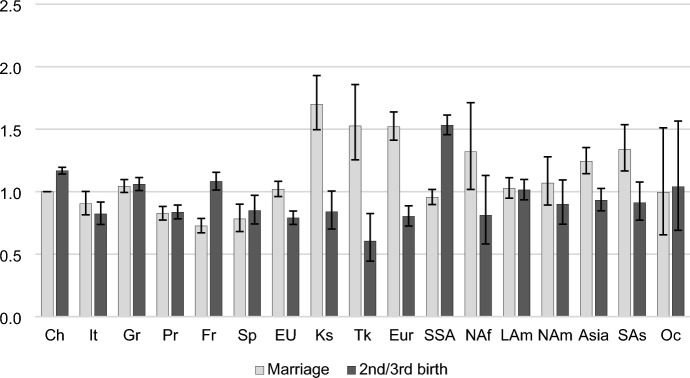
Outcomes of unmarried mothers: Relative risks of a second/third birth or first marriage by migrant origin. *Note*: *CH* Switzerland, *It* Italy, *Gr* Germany, *Pr* Portugal, *Fr* France, *Sp* Spain, *EU* other EU countries, *Ks*  Kosovo, *Tk* Turkey, *Eur* other European countries (not in the EU), *SSA* Sub-Saharan Africa, *NAf* North Africa, *Lam* Latin America, *Nam* North America, Asia, *SAs* South Asia, *Oc* Oceania. Control variables: age, time since last birth, employment status, household income. *Sources*: Statpop, Bevnat, CdC (2012–2018). Authors’ own calculations. 95% confidence intervals

We present the results of the piecewise constant exponential models for unmarried childless women (Fig. 2) and unmarried mothers with one or two children (Fig. 3). Relative risks are the result of an interaction between the type of event and migrant origin in a competing events framework. Unmarried women, who may be unpartnered or living in a nonmarital cohabitation, are at risk of getting married or having a(n additional) child. The reference category is the hazard of marrying among unmarried Swiss women (denoted by 1). Because distinct arrival effects are expected mainly for the first birth, we present the results separately for newly arrived and settled immigrants in the main text for childless women and in the Appendix for higher order births. Figure 2a shows the relative risks of a first birth or first marriage among childless unmarried women. In this population, the risk of marrying is the highest, followed by the risk of a first birth. Among Swiss women, the risk of marrying is about twice as high as the risk of having a first birth. Overall, we find similar patterns among women from EU countries. Compared to the first birth risk among natives, the risk of a first birth out of wedlock is somewhat higher for all EU groups, ranging from a 10% increase among women from other EU countries to a 75% increase among French women. Portuguese women stand out by an even higher risk of first birth (2.7 times higher than the risk of a first birth among natives), but also by a higher propensity to marry (30% higher than the risk of marrying among natives).

Greater differences emerge among women from non-EU countries. The most prominent difference is observed among women from Sub-Saharan Africa for whom the risk of a first birth outside of marriage is the highest among all groups; their propensity to marry is, however, comparable to that of native women. Part of this dynamic can be explained by the very nature of the administrative data, which only records civil statuses (as compared to, for example, religious marriages). Contrary to expectations, women from non-EU countries have similar or even higher first birth risks than unmarried Swiss women. Only women from Turkey and North America have a lower risk of first birth outside marriage. Other patterns of family formation marked by a higher propensity to marry can be identified. This is the case among women from Kosovo, Turkey, and other European countries, and to a lesser extent for women born in North Africa, Latin America, and South Asia.

The results clearly show distinct family behaviours for newly arrived immigrants compared to settled immigrants (Fig. 2b). We find that newly arrived immigrant women from EU countries have similar first birth risks to Swiss women (with the exception of the Portuguese). In contrast, settled EU immigrants have a higher risk of a first birth compared to Swiss women Marriage risksdo not differ between newly and settled immigrants, again, with the exception of the Portuguese whose risk of marrying is elevated following immigration.

The relationship between family events and immigration operates differently among non-EU migrants. Women from Kosovo, Sub-Saharan Africa, and South Asia have increased first birth risks shortly after arrival (as compared to Swiss women, and to their counterpart who spent more time in the country). For other groups, this association is reversed. This is the case among women from Latin and North America for whom the risk of a first birth is higher after two years. Marriage risks are higher among women from Kosovo, Sub-Saharan Africa and Latin America in the first two years following immigration. Higher birth and marriage risks in the first years are consistent with family patterns in the context of marital migration among partnered unmarried women.

Figure 3 shows the relative risks of a birth or marriage among unmarried mothers, providing a measure of the extent to which nonmarital childbearing continues beyond the first birth. Due to a smaller number of events (only 1% of all births are a third birth of unmarried mothers) and large confidence intervals for some groups, we have pooled the transitions to second and third births. Differences in marriage and birth risks between migrant groups and natives are much smaller among unmarried mothers than they were among childless unmarried women. Unmarried mothers are a select group, and probably even more so among women born in countries with more conservative family values; the results reflect this dynamic.

Again, the results show greater similarities to the patterns of native women among women born in EU countries. The Portugueses, French, and Spaniards are somewhat less likely than natives to marry; other groups do not differ. Swiss natives and French women are the most likely to have a second or third child as unmarried and these are the only European groups who are more likely to have a second or third child than to marry. Non-EU women also show lower second or third birth risks compared to natives. Again, women from Sub-Saharan Africa stand out with 30% higher risks of a second or third birth while unmarried. By contrast, Turkish unmarried women are the least likely to give birth to a second or third child. Among unmarried mothers, some groups maintain a high propensity to marry. This is the case among women born in Kosovo, Turkey, other European countries, North Africa, Asia, and South Asia.

### Outcomes of Married Women

**Fig. 4:**
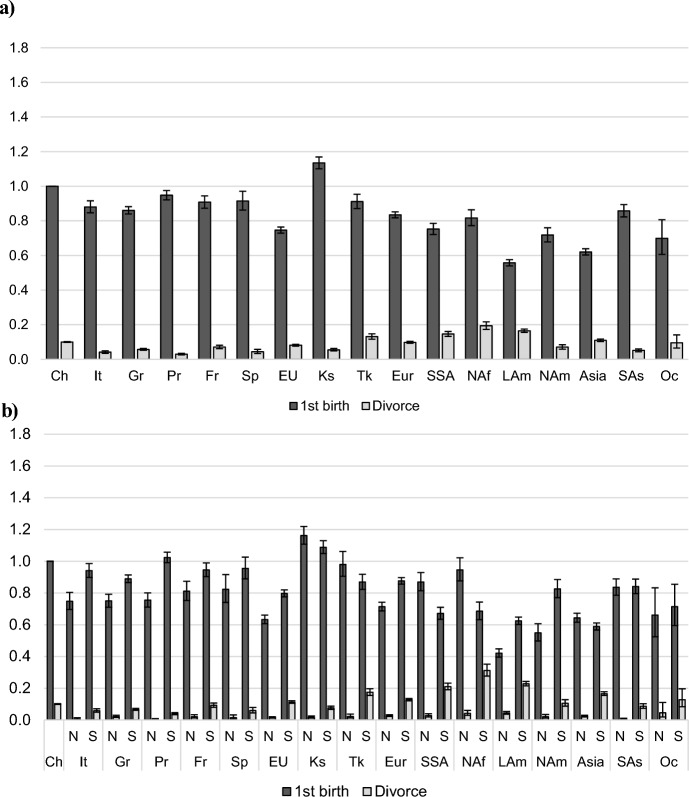
Outcomes of childless married women. **a** Relative risks of a first birth or divorce by migrant origin. **b** Relative risks of a first birth or divorce by migrant origin, separately for newly arrived immigrants and settled immigrants. *Note*: *CH* Switzerland, *It* Italy, *Gr* Germany, *Pr* Portugal, *Fr* France, *Sp* Spain, *EU* other EU countries, *Ks* Kosovo, *Tk* Turkey, *Eur* other European countries (not in the EU), *SSA* Sub-Saharan Africa, *NAf* North Africa, *Lam* Latin America, *Nam* North America, Asia, *SAs* South Asia, *Oc* Oceania. Control variables: age, marriage duration, employment status, household income. *N* Newly arrived immigrants (first two years in the country), *S* Settled immigrants (subsequent years). *Sources*: Statpop, Bevnat, CdC (2012–2018). Authors’ own calculations. 95% confidence intervals

**Fig. 5:**
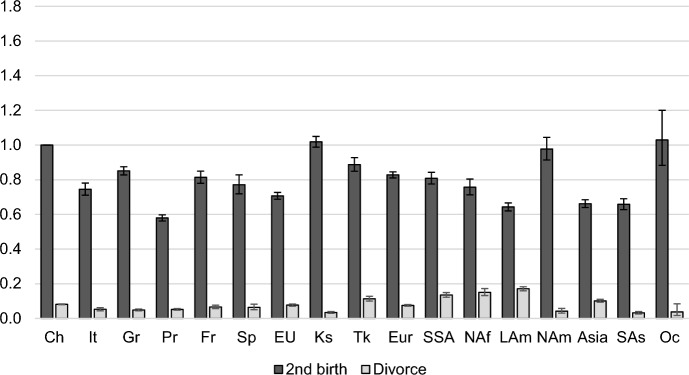
Outcomes of married women with one child: Relative risks of a second birth or divorce by migrant origin

**Fig. 6:**
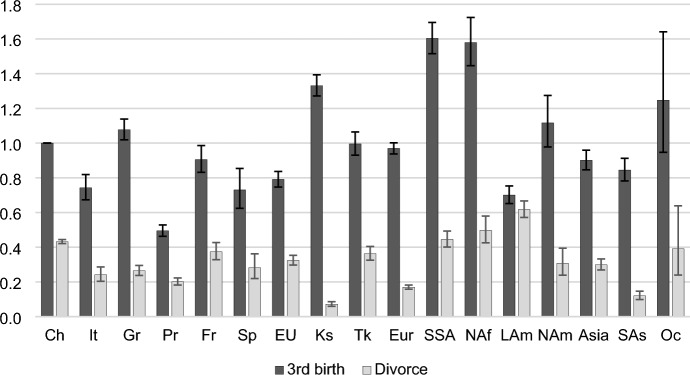
Outcomes of married women with two children: Relative risks of a third birth or divorce by migrant origin. *Note*: *CH* Switzerland, *It*  Italy, *Gr* Germany, *Pr* Portugal, *Fr* France, *Sp* Spain, *EU* other EU countries, *Ks* Kosovo, *Tk* Turkey, *Eur* other European countries (not in the EU); *SSA* Sub-Saharan Africa, *NAf* North Africa, *Lam* Latin America, *Nam* North America, Asia, *SAs* South Asia, *Oc* Oceania. Control variables: age, marriage duration, time since last birth, employment status, household income. *Sources*: Statpop, Bevnat, CdC (2012–2018). Authors’ own calculations. 95% confidence intervals

Figures 4, 5, and 6 illustrate the patterns of married women's transitions to divorce or a first, second, and third childbirth, respectively. The reference category is the hazard of a (first, second, or third) birth among married Swiss women (denoted by 1). Among childless married women (Fig. 4), childbirth is the most likely outcome, followed by divorce; the latter being about ten times less likely than the former.

Married women show some variation in first birth risks between migrant groups. Women from the EU countries have slightly lower first birth risks (10% to 25% lower). First birth risks of non-EU migrants are generally somewhat lower than those of native women. Women from Latin America and Asia have the lowest first birth risks; about half of that of Swiss women. The only exception is women form Kosovo whose first birth risks are about 15% higher than those of native women.

Just like the transition to a first birth among unmarried women, newly arrived married women from EU countries have lower first birth risks than settled immigrant women in the same family situation. After a settlement period of more than 2 years, the hazard of a first birth is higher and more similar to those of Swiss women. Nevertheless, women from Germany and other EU countries maintain lower first birth risks than natives in the long run; about 10% and 20%, respectively. Women from non-EU countries, by contrast, often experience increased risks of the transition to a first birth shortly after immigration compared to settled immigrants. This is the case for women from Kosovo, Turkey, Sub-Saharan Africa, and North Africa. This pattern is reversed for some non-EU groups: women from Latin and North America, and women from other European countries are more likely to have a first child after more than 2 years in the country.

We also find differences in the magnitude of divorce risks by migrant origin. We find more differences in the risks of divorce among women from non-EU countries with women from Kosovo, South Asia, and North America having the lowest divorce rates and women from North Africa, Sub-Saharan Africa, and Latin America having the highest. Divorce risks are especially low among newly arrived immigrants for both EU and non-EU groups.

Figure 5 shows the patterns of transitions to a second birth vs. divorce among married mothers with one child. Variations between migrant groups for the risk of a second birth are comparable to those for the risk of a first birth. Women from all EU countries have lower second birth risks than native Swiss women with the Portuguese having the lowest risks. Only women from Kosovo, North America, and Oceania have second birth rates comparable to those of native women; all other groups are less likely to have a second birth. The relative risks of divorce, on the other hand, are very similar to those observed for childless married women.

Figure 6 shows the relative risks of a third birth or divorce among married mothers with two children. Compared to the patterns of transitions to a first and second birth, significant differences emerge between groups. Among EU countries, only women from Germany display slightly higher third birth risks (about 10%) than Swiss women. While Portuguese women have about half the risk of Swiss women, other groups are about 10% (France) to 25% (Italy, Spain) less likely to have a third birth. Many migrant women from non-EU countries have high third birth rates. Immigrants from Kosovo, Sub-Saharan Africa, and North Africa are more likely than native married women to have a third child. The risks increase by 30% for the first group and by 60% for the last two groups.

Women from Latin America have the highest divorce rates and women from Sub-Saharan Africa and North Africa have similar divorce rates compared to native women. All other groups are less likely than native women to get divorced among married mothers with two children.

### Outcomes by Linguistic Regions

**Fig. 10:**
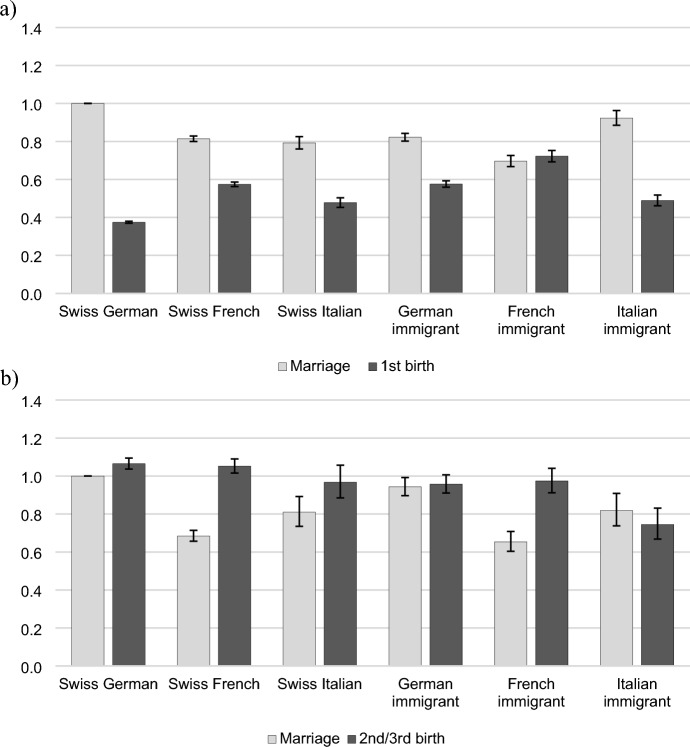
Outcomes of unmarried women. **a** Relative risks of marriage of a first birth by linguistic region and migrant origin. **b** Relative risks of marriage or a second or third birth by linguistic region and migrant origin. *Sources*: Statpop, Bevnat, CdC (2012–2018). Authors’ own calculations. 95% confidence intervals

**Fig. 11:**
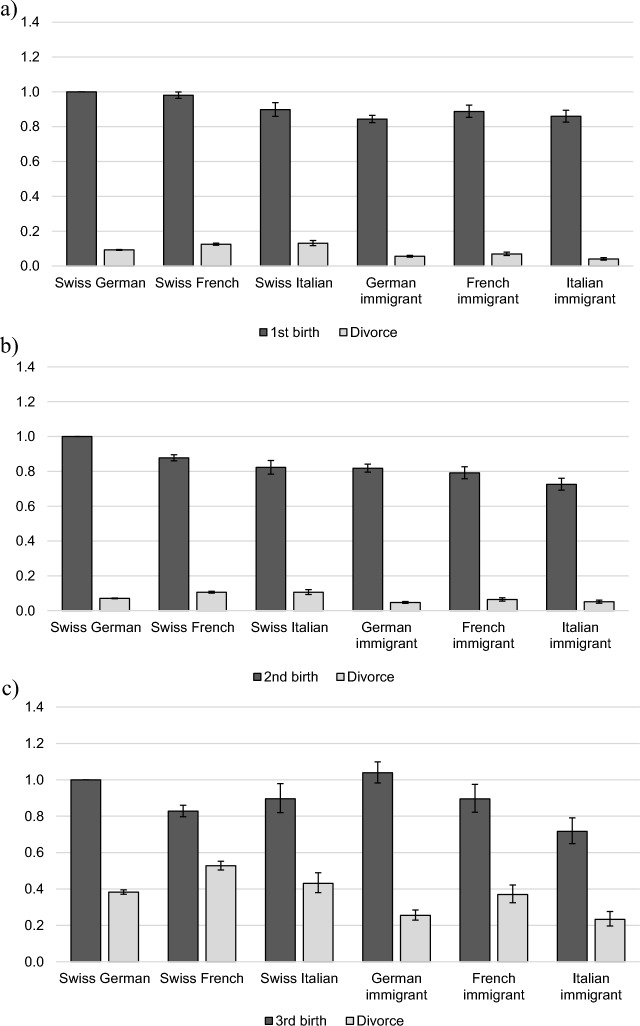
Outcomes of married women. **a** Relative risks of a first birth or divorce by linguistic region and migrant origin. **b** Relative risks of a second birth or divorce by linguistic region and migrant origin. **c** Relative risks of a third birth or divorce by linguistic region and migrant origin. *Sources:* Statpop, Bevnat, CdC (2012–2018). Authors’ own calculations. 95% confidence intervals

We further explore the specific behaviours patterns of immigrants from neighbouring countries with that of natives from respective linguistic regions. Results (See Figs. 10 and 11 in appendix) show both within and between group differences. For the outcomes of childless unmarried women, German and French immigrants display less conservative family behaviours: marriage rates are lower and first nonmarital births higher than among their native counterparts. German and French immigrants are over-represented among highly skilled workers and, as a result, are likely to differ from their origin country counterparts in terms of demographic characteristics and unobserved attitudes to work and family. However, for the transition to higher order births among unmarried mothers, the transitions of French (German) immigrants practically mirror those of native French (German); Italian immigrants by contrast have lower birth rates than their Swiss Italian counterparts. For the outcomes of married mothers, transition to a third birth appeared higher for both German immigrants and German-speaking natives. These results bring nuances to the heterogeneity of native populations and the need for a more in-depth assessment of the interaction between culture and the role played by immigration in family processes.

## Discussion

This paper simultaneously analysed marriage and childbearing changes among native and immigrant women in Switzerland. Taking advantage of full-population registers, we analysed detailed patterns of transitions by migrant’s country of origin distinguishing newly arrived immigrants from settled immigrants. The paper contributes to the literature by providing new insights into the partnership context of fertility, and the interrelatedness of family and migration dynamics overall and in Switzerland in particular. Childbearing in and outside of marriage (whether by lone parents or consensual partners) has been seen as a manifestation of the Second Demographic Transition; a development induced by ideational and cultural transformations (Lesthaeghe, 2010, 2014). However, studies show that assimilation in the family sphere among immigrant populations occurs more slowly than assimilation in other domains leading to discussions on a possible Third Demographic Transition (Coleman, 2006).

Using a multistate event history approach, we found that most of the differences in family formation patterns between migrant groups and natives were in the sequencing of marriage and first birth among childless unmarried women. Immigrants from countries with more conservative family systems experienced marriage-centred family behaviours (e.g. Turkey). Even when having a first child outside of marriage, these groups maintained a higher propensity to marry later. Transition to a first birth outside marriage was most frequent among immigrants from countries where this family behaviour is more widespread (France, Portugal, Spain). This is in line with the socialisation hypothesis. Nevertheless, the results revealed a great deal of complexity and specificity among migrant groups that goes beyond socialisation.

In fact, we found trends of nonmarital family formation among both EU and non-EU groups albeit with very different timing around immigration. When they were unmarried, migrant women were generally more likely than Swiss women to have a first birth (with a few exceptions). One explanation lies in the composition of the unmarried population, which includes both individuals in consensual partnerships and unpartnered individuals. The native population includes women at different stages of their life course. By contrast, non-EU groups for whom entry requirements are often conditional on family visas often migrate at specific stages in their family and reproductive lives. The fact that first childbirth outside marriage is most prevalent only in the first two years after immigration suggests that these transitions are induced by the migration process; a matter of timing and selection.

The risks of a second or third birth outside marriage were highest for natives, as well as for immigrants from France and Sub-Saharan Africa suggesting that out of wedlock motherhood trajectories are a more sustainable alternative to marriage among these groups. As mentioned, however, the marital behaviour of women from sub-Saharan African countries likely reflects a lack of registered civil marriages and the prevalence of traditional or religious marriages; a trajectory that differs from that of SDT. One must also consider that women who migrate as single are a select group and their family preferences are likely to differ from those of their married counterparts. The descriptive statistics showed that some non-EU groups are very unlikely to be unmarried (at arrival). By contrast, EU migrants are more represented in the unmarried category and are also more likely to experience childbearing in this situation, suggesting that nonmarital family formation is a common experience among this group. Once married, patterns of transitions are more similar across groups. It is the risk of a third birth that show marked differences between migrant groups; first and second birth risks are somewhat lower but similar across groups.

Distinguishing between newly arrived migrants and settled migrants allowed for a better understanding of the rationale behind immigration and family formation, and interdependency of these events. Migration policies influence both family choices (consensual unions versus marriages) and the profile of migrant populations (i.e. in terms of family and professional aspirations). We found timing effects among childless (married and unmarried) women, but no clear patterns emerged among mothers. There was a clear difference in the likelihood of having a first child or getting married in the first two years following immigration compared to subsequent years. More importantly, we found opposite arrival effects for EU and non-EU migrants which points to different mechanisms associated with the immigration process.

Newly arrived EU migrants were less likely to have a first birth than settled EU migrants. This may be explained by the fact that most EU migrants come to Switzerland for professional reasons. Many of them migrate as primary migrants and are unpartnered. Besides, the early years may be seen as an investment in the professional sphere and therefore not considered an appropriate time to start a family even for those in a relationship. This is in line with the disruption hypothesis. Nevertheless, selection effects are also likely at play. Women who migrate for professional reasons may be more inclined to prioritise the professional sphere over the family sphere (aspirations for the latter may also be lower among these migrants). The trade-off between family and work in the country remains important for women given the high costs and low provision of childcare support, thus reinforcing this dynamic. In addition, migration to Switzerland by EU migrants is often temporary. Many will only stay for a few years to gain work experience and consolidate their finances—a life course stage that might not be seen as compatible with family formation.

On the contrary, newly arrived non-EU migrants had higher transition rates to marriage and first birth compared to their settled counterparts (especially among married women). This is in line with previous studies documenting the ‘3 pack’ of marriage, migration, and first child (Milewski, 2007), thus supporting the interrelation of events hypothesis. Although some migrants may have liberal views on cohabitation and marriage, legal constraints certainly reinforce the link between marriage and migration. This requirement may explain the higher propensity to marry for some groups, over and above individual preferences, especially non-Europeans. Nevertheless, studies document strong social reproduction of family formation behaviours among the second generation (see, for instance, Mikolai & Kulu, 2022) suggesting that legal requirements alone do not explain conservative attitudes towards marriage. The higher risks of marriage and third births for some migrant groups also support our hypothesis that non-EU migrant women are positively selected for their family aspirations. Non-EU nationals who wish to migrate for professional reasons face many obstacles. Nonetheless, some non-EU groups are likely over-represented among the highly skilled. Immigrants from North America are a good example as they often migrate to Switzerland for a brief period to take on a specific appointment.

Switzerland has a comparatively conservative attitude towards marriage and marital childbearing and many EU migrants come from countries that are further along in the Second Demographic Transition. As expected, we found homogeneous transition patterns among EU migrants with nonmarital family formation being common. By contrast, we found greater heterogeneity among migrants from non-EU countries; a much more diverse population in terms of cultural background and migration process. When comparing the family formation patterns of immigrants from neighbouring countries with that of natives from respective linguistic regions we found both within and between group differences. Research rarely takes into account the heterogeneity of native populations and the differentiated assimilation of immigrants into specific native subcultures. We see these lines of research as a mean to enrich discussions on the interplay between culture and migration processes. More importantly, this study showed how migration policies (through specific family migration channels and demand for labour migrants) selects a specific demographic group in the countries of origin and how this, in turn, attenuates or reinforces the heterogeneity of family behaviours. Selection looms large in this line of research and there is a need to thoroughly address the role played by migration policies when interpreting differentiated demographic behaviours of immigrants. Future research would also benefit from analysing duration within different states (including cohabitation) as a way to disentangle for whom nonmarital cohabitation and childbearing become a sustainable family pathway as opposed to a temporary stage before marriage, thus enriching the discussion of the role immigrants play in the Second Demographic Transition and family change in Europe.

## Figures and Tables

**Fig. 7:**
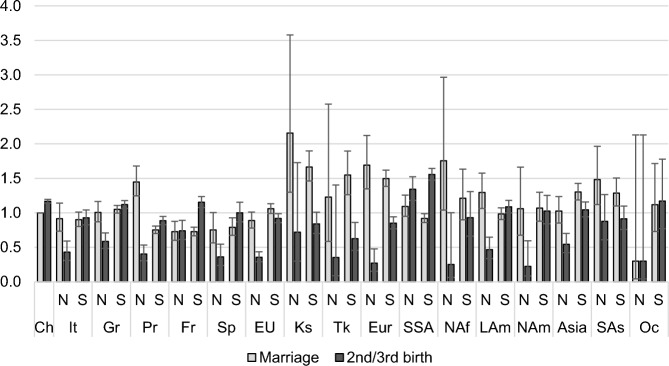
Outcomes of unmarried mothers: relative risks of a second/third birth or first marriage by migrant origin, separately for newly arrived immigrants and settled immigrants

**Fig. 8:**
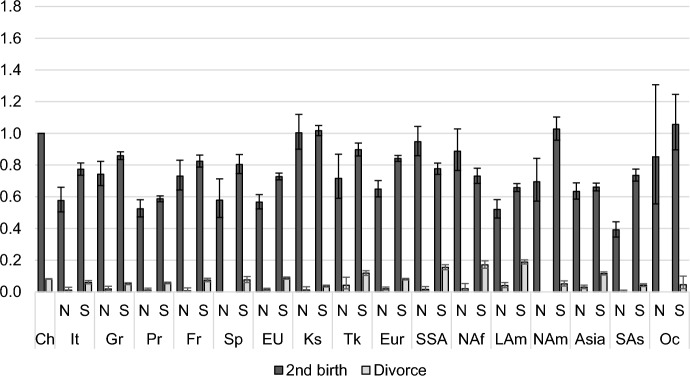
Outcomes of married mothers: relative risks of a second birth or divorce by migrant origin, separately for newly arrived immigrants and settled immigrants

**Fig. 9:**
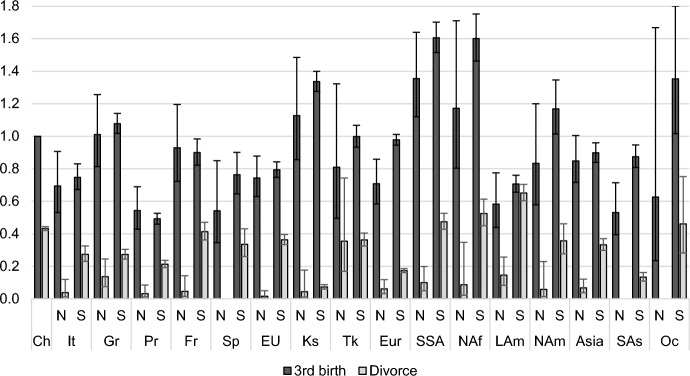
Outcomes of married mothers: relative risks of a third birth or divorce by migrant origin, separately for newly arrived immigrants and settled immigrants. *Note: CH* Switzerland, *It* Italy, *Gr* Germany, *Pr* Portugal, *Fr*  France, *Sp* Spain, *EU* other EU countries, *Ks* Kosovo, *Tk* Turkey, *Eur* other European countries (not in the EU), *SSA* Sub-Saharan Africa, *NAf* North Africa, *Lam* Latin America, *Nam* North America, Asia, *SAs* South Asia, *Oc* Oceania. *N*  Newly arrived immigrants (first two years in the country), *S* Settled immigrants (subsequent years). *Sources*: Statpop, Bevnat, CdC (2012–2018). Authors’ own calculations. 95% confidence intervals

## Data Availability

The data that support the findings of this study are available from the Swiss federal statistical office but restrictions apply to the availability of these data, which were used under license for the current study, and so are not publicly available.

## Appendix

See Tables 2 and 3, Figs. 7, 8, 9, 10, and 11.

**Table 2:** Distribution of independent variables by migrant origin

	Ch	It	Gr	Pr	Fr	Sp	EU	Ks
*Age group*								
15–19	32.3	11.1	8.7	14.0	12.9	9.6	9.1	12.8
20–24	16.8	16.6	14.2	14.3	17.7	14.6	17.1	33.2
25–29	14.8	21.2	25.3	20.4	24.1	21.4	23.6	26.3
30–34	12.9	18.5	23.5	18.8	19.7	21.4	21.6	14.6
35–39	11.2	16.7	15.4	16.6	14.1	18.7	16.4	8.1
40–44	12.1	15.9	12.9	16.0	11.5	14.3	12.3	5.1
*Marital status*								
Unmarried	75.7	64.3	72.4	45.0	73.1	65.3	62.3	24.7
Married	24.4	35.7	27.6	55.0	26.9	34.7	37.7	75.4
*Children*								
0	76.7	70.3	76.3	50.1	73.2	67.8	71.6	52.5
1	9.8	13.9	12.9	26.6	13.6	15.8	14.6	21.1
2 or more	13.5	15.9	10.8	23.2	13.3	16.4	13.8	26.3
*Employed*								
Yes	31.7	33.6	22.9	22.7	29.6	33.5	34.6	41.4
No	68.4	66.5	77.1	77.3	70.4	66.5	65.4	58.6
*Household income (CHF)*								
0–50,000	13.3	30.9	21.4	15.8	26.2	25.3	29.7	17.9
50,000–100,000	25.2	29.1	25.3	37.5	23.2	27.5	24.3	39.4
100,000–150,000	28.7	19.0	23.0	32.4	19.2	19.3	18.0	24.3
150,000 +	32.8	21.1	30.4	14.3	31.4	27.9	28.0	18.4
Total population	1,058,801	40,582	102,927	64,832	48,271	16,734	115,533	24,306

	Tk	Eur	SSA	NAf	LAm	NAm	Asia	SAs	Oc
*Age group*									
15–19	8.2	8.6	17.8	9.4	14.4	21.9	14.3	8.0	19.1
20–24	11.2	18.8	17.7	12.4	11.9	18.6	19.5	13.6	14.7
25–29	20.2	24.7	21.1	20.8	17.7	17.2	18.4	26.3	16.3
30–34	22.8	20.2	18.3	23.0	21.1	17.3	19.0	23.8	18.5
35–39	19.7	15.1	13.9	18.9	19.3	13.1	15.8	16.1	16.6
40–44	18.0	12.6	11.3	15.5	15.6	12.0	13.1	12.2	14.9
*Marital status*									
Unmarried	22.9	29.1	58.2	27.5	42.3	64.4	48.1	28.9	61.2
Married	77.1	70.9	41.8	72.5	57.7	35.6	51.9	71.1	38.8
*Children*									
0	42.7	49.7	59.8	56.8	61.5	77.4	68.8	51.2	72.8
1	22.1	21.2	22.3	22.5	21.4	10.1	17.1	21.8	11.0
2 or more	35.2	29.1	17.9	20.7	17.1	12.5	14.1	27.0	16.1
*Employed*									
Yes	38.1	34.2	50.2	52.5	45.0	57.2	59.3	56.5	50.4
No	61.9	65.8	49.8	47.5	55.0	42.8	40.8	43.5	49.6
*Household income (CHF)*									
0–50,000	29.9	18.9	48.9	37.6	27.1	32.1	32.1	36.4	28.8
50,000–100,000	37.2	32.9	22.5	30.1	29.1	16.5	21.1	29.8	16.2
100,000–150,000	20.3	26.7	13.0	15.4	21.2	14.4	16.5	17.2	17.1
150,000 +	12.5	21.5	15.7	16.9	22.6	37.0	30.3	16.7	37.9
Total population	18,690	93,361	35,159	11,232	55,429	20,053	70,799	24,561	3,341

*Sources*: Statpop, Bevnat, CdC (2012–2018)

*CH* Switzerland, *It* Italy, *Gr* Germany, *Pr* Portugal, *Fr* France, *Sp* Spain, *EU* other EU countries, *Ks* Kosovo, *Tk* Turkey, *Eur* other European countries (not in the EU), *SSA* Sub-Saharan Africa, *NAf* North Africa, *Lam* Latin America, *Nam* North America, Asia, *SAs* South Asia, *Oc* Oceania

**Table 3:** Hazard ratios of covariates by marital status and parity

	Unmarried childless	Unmarried with 1 or 2 children	Married childless	Married with 1 child	Married with 2 children
*Age group*					
15–19	1.00	1.00	1.00	1.00	1.00
20–24	4.31***	3.95***	1.03	1.15	1.27
25–29	10.93***	5.77***	1.17***	1.29**	1.26
30–34	14.11***	5.76***	1.19***	1.21**	0.97
35–39	8.76***	4.21***	0.80***	0.85*	0.53
40–44	2.57***	1.84***	0.24***	0.28***	0.22***
*Time since last birth*					
0–1 year		1.00		1.00	1.00
1–3 years		1.08***		3.35***	0.06***
3–5 years		0.59***		2.11***	0.05***
5+ years		0.32***		1.22***	0.04***
*Marriage duration*					
0–1 year			1.00	1.00	1.00
1–3 years			0.55***	0.70***	0.57***
3–5 years			0.42***	0.66***	0.66***
5+ years			0.31***	0.53***	0.50***
*Employed*					
No	1.00	1.00	1.00	1.00	1.00
Yes	1.35***	1.02	1.22***	1.16***	1.18***
*Household income (CHF)*					
0–50,000	1.00	1.00	1.00	1.00	1.00
50,000–100,000	1.10***	1.24***	1.10***	1.00	0.63***
100,000–150,000	1.81***	1.43***	1.23***	1.04***	0.47***
150,000+	1.79***	1.41***	1.34***	1.07***	0.51***
Constant	0.00***	0.01***	0.23***	0.10***	0.06***
N	8,982,266	991,892	1,713,800	1,900,696	2,800,072

*Sources*: Statpop, Bevnat, CdC (2012–2018). Authors’ own calculations. 95% confidence intervals

*CH* Switzerland, *It* Italy, *Gr* Germany, *Pr* Portugal, *Fr* France, *Sp* Spain, *EU* other EU countries, *Ks* Kosovo, *Tk* Turkey, *Eur* other European countries (not in the EU), *SSA* Sub-Saharan Africa, *NAf* North Africa, *Lam* Latin America, *Nam* North America, Asia, *SAs* South Asia, *Oc* Oceania

## Footnotes

1 National statistics in Switzerland are compiled on the basis of citizenship rather than the country of birth.

2 The population register was first introduced in 2010 but it is only since 2012 that a household ID is available.

3 Based on the number of children in the household and their ages we were able to infer parity with a high degree of certainty. We cross-validated this measure (1) using the Structural Survey, a nationally representative survey that contains information on the relationships between household members and (2) using the parity variable in the Vital register (for those having children between 2013–2018).
